# Evaluation of an interpretable deep-learning model for the automated plan review of intensity-modulated radiation therapy

**DOI:** 10.3389/fonc.2026.1823869

**Published:** 2026-07-15

**Authors:** Yuhan Fan, Jiawen Shang, Ke Zhang, Zhihui Hu, Zhiqiang Liu, Hui Yan, Peng Huang

**Affiliations:** 1 Department of Radiation Oncology, National Cancer Center/National Clinical Research Center for Cancer/Cancer Hospital, Chinese Academy of Medical Sciences and Peking Union Medical College, Beijing, China; 2 State Key Laboratory of Cardiovascular Disease, Fuwai Hospital, National Center for Cardiovascular Disease, Chinese Academy of Medical Sciences and Peking Union Medical College, Beijing, China

**Keywords:** anomaly, autoencoder, deep learning, intensity modulated radiation therapy, model interpretation, quality assurance

## Abstract

**Background:**

Intensity-modulated radiation therapy (IMRT) is a treatment modality for delivering a higher radiation dose to the target using multiple radiation beams from various gantry angles. Since an IMRT plan is complex and presents a high risk, quality assurance is crucial. In routine clinical practice, a radiotherapy treatment plan review is manually conducted by experienced planners to ensure the high quality and reliability of an IMRT plan. To assist this procedure, several machine-learning methods have been developed to automatically identify outliers or anomalous plans from a large amount of treatment plans.

**Purpose:**

Due to the highly complex and non-linear nature of most machine-learning methods, their results are less explainable, and therefore, cannot be fully trusted by clinical users. To alleviate this issue, an unsupervised deep-learning model with an interpretable tool was introduced and evaluated on our clinical database.

**Methods:**

Six hundred IMRT treatment plans were collected from our institute and the relevant features were extracted. A standard autoencoder (AE) was used to build an anomaly detection model from the normal plans and identify anomalies with exceptionally high reconstruction errors. To help explain the model output, a feature perturbation interpretation (FPI) method was introduced to rank the impacts of the features on the detected anomalies. To validate the effectiveness of the FPI method, the feature ranks provided by the local depth-based isolation forest feature importance (Local-DIFFI) and SHapley Additive exPlanations (SHAP) methods were compared. In addition, the effectiveness of the FPI method was evaluated on the other four classic detection models, local discrete factor (LOF), density-based spatial clustering of applications with noise (HDBSCAN), one class of support vector machines (OC-SVM), and principal component analysis (PCA).

**Results:**

The AE achieved the best detection performance among all the methods. The area under the curve (AUC) value of the AE was 0.98, and the average accuracy, precision, and F1 score were 0.91, 0.61, and 0.74, respectively. The top five features with the largest impacts on the detected anomalies obtained by FPI, local-DIFFI, and SHAP are the same.

**Conclusion:**

The AE is an effective deep-learning model in identifying anomalous plans compared to classic detection models. The FPI method is universal and a reliable tool in determining the feature impact on the anomalies. Their combination provided a highly interpretable deep learning model for automatic plan review in radiotherapy.

## 1 Introduction

Cancer patients now receive radiotherapy for neo-adjuvant, definitive, adjuvant, or palliative purposes (1–3). As a major treatment modality, intensity-modulated radiation therapy (IMRT) delivers a higher radiation dose to the target in patient while sparing the surrounding healthy tissues. A strengthened quality-control process, such as a physics plan review, has been enforced to ensure the quality and safety of treatment plans (4–6). A full physics plan review for IMRT includes parameter checking related to diagnosis, prescription, planning, and field-specific settings (7, 8). It is time-consuming and relies highly on the experience of the reviewing physicist (9).

Automated methods have been gradually used to assist plan review processes in a radiotherapy clinic and can handle various tasks including a quality-control plan (10), a data comparison plan (11), a physics chart review (12), and an integrity-checking plan (13). Recently, deep learning has been introduced in radiotherapy. Several applications have been explored including treatment plan quality control (14), monitoring the mechanical status of the treatment machine (15), patient-specific quality assurance (PSQA) (16–18), and plan review and checking. Unsupervised deep-learning models, such as autoencoder (19–21), have also been employed to assist with anomaly detection during the plan review process. The result is encouraging but lacks interpretability, which hinders its clinical use.

Artificial intelligence (AI) models are now widely adopted in clinical practice (22). It is crucial to make them explainable so that healthcare providers can understand the reasoning process behind the model output (23). To address this issue, interpretation methods have been developed to offer insights into the decision-making process of AI models (24). Models like decision trees or linear regression are inherently interpretable but may lack complexity (25). Techniques such as SHAP (SHapley Additive exPlanations) (26) and LIME (local interpretable model-agnostic explanations) (27) provide insights into model predictions after training. However, both of these methods are computationally expensive (28). Local-DIFFI (local depth-based isolation forest feature importance) is an efficient method to evaluate feature contribution to the result but specified for isolation forest (IF) (29). To make this idea feasible to most AI models, the feature perturbation interpretation (FPI) method was developed by systematically changing each of the features and observing its effect on the model output (30).

In this study, a framework using an AE model and perturbation-based tool for anomaly detection in a physics plan review was investigated. First, the relevant features were extracted from radiotherapy plans. Then, an AE model was built on the features collected from the normal plans. Finally, the AE model was tested on the dataset consisting of normal and anomalous plans. The sample with an exceptionally high reconstruction error was determined as anomaly. The FPI method was used to rank the impact of the features on detected anomalies. To evaluate its effectiveness, the result provided by the FPI method was compared with those provided by local-DIFFI and SHAP methods, respectively.

## 2 Materials and methods

### 2.1 Plan review

In our clinic, radiotherapy plans are first examined using a plan-checking software and then manually verified by senior medical physicists. The dose prescription for the target, the number of fractions, dose constraints, and others, for critical organs are checked first. Next, the plan parameters, such as beam and collimator angles, are checked to ensure their feasibility and safety. If there were improper settings, such as unconventional beam configuration, the plan is put on hold for further revision. The machine learning-based detection algorithm is then implemented in the plan-checking software. The plan parameters are extracted from the oncology information system and used as input to the detection model, which calculates the anomaly score. If the score is higher than a preset threshold, this plan is labeled by a warning sign.

For classification, the treatment plans can be categorized into three groups: normal, error, and anomalous (or outlier). The normal plans are those that strictly follow clinical guidelines, maximally satisfy clinical requirements, and can be delivered without potential issues. The error plans are those that apparently violate clinical guidelines, regulations, and tolerances. The anomalous plans (or outliers) are those that follow clinical guidelines but have unconventional parameter settings. These anomalous plans include unconventional beam configuration, uncommon optimization constraints, unexpected dose distributions, and improper leaf positions. For example, two beams are too close, which could cause decreased modulation of their intensity maps. Multi-collimator leaves have a certain distance from the target, which could result in a higher dose to the surrounding normal tissues.

### 2.2 Data

The dataset was collected in our institute between 2016 and 2022. The dataset comprises 576 radiotherapy plans. Each plan consists of two tangent conformal fields and two intensity-modulated radiation fields. Among them, 558 and 18 plans are normal and anomalous instances, respectively. A total of 30 relevant features were extracted from each plan (**Table 1**). The first column lists the names of the eight feature groups and the third column lists the number in each feature group. The descriptions of the 30 features are detailed in Appendix 1. All categorical features were transformed into numerical values via one-hot encoding method. Min–max normalization was applied to rescale the numerical features to a range of [0–1].

**Table 1:** The features extracted from each IMRT plan.

Name	Description	Elements	Type	Unit
Segment	Segments per intensity-modulated field	2	Integer	Number
SSD	Source-to-skin distance per field	4	Float	cm
Coll_x1_	Collimator position in the x1 direction per field	4	Float	cm
Coll_x2_	Collimator position in the x2 direction per field	4	Float	cm
Coll_y1_	Collimator position in the y1 direction per field	4	Float	cm
Coll_y2_	Collimator position in the y2 direction per field	4	Float	cm
Gantry	Gantry angle per field	4	Integer	degree
MU	Monitor unit (MU) per field	4	Float	MU

This study was conducted in accordance with the Declaration of Helsinki (as revised in 2013). It was approved by the Ethics Committee of the National Cancer Center/Cancer Hospital, Chinese Academy of Medical Sciences, and Peking Union Medical College (No. NCC2023C-675). The requirement for a written informed consent was waived due to the retrospective design of the study. The data presented on this study can be made available upon request. Approval from a research ethics committee may be required.

### 2.3 Anomaly detection

**Figure 1:**
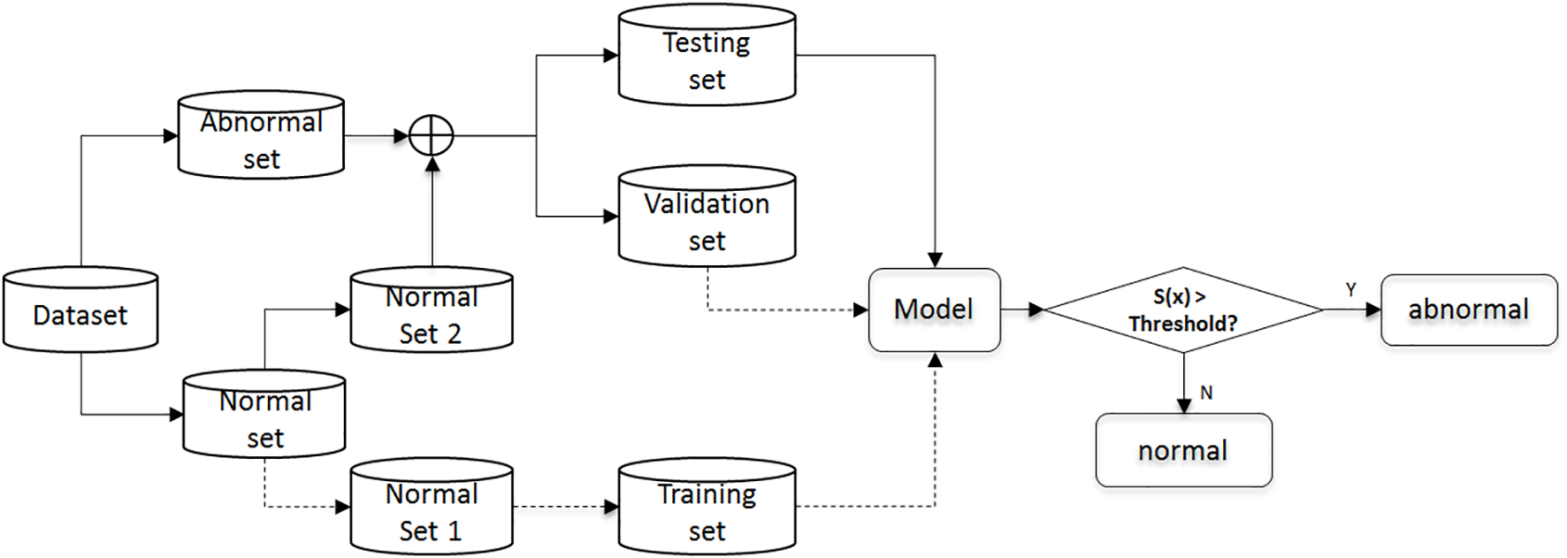
The workflow of the anomaly detection process in a physics plan review.

Anomaly detection is designed to detect outliers using the machine-learning method. The workflow adopted in this study is shown in **Figure 1**. All samples were divided into normal and abnormal sets. The majority (95%) of the normal plans were allocated to Normal Set 1 and the remainder (5%) was allocated to Normal Set 2. The Normal Set 1 was used for model training. Note that the dotted line in **Figure 1** indicates the training flow of the model training. The Normal Set 2 and the Abnormal Set were used for model validation and testing following a ratio of 1:2. For a sample *x*, the detection model calculated its anomaly score *S(x)*. *S(x)* is the decision function of the model in quantifying its deviation from the normal plan. An exceptionally high score indicates a significant deviation from the normal instance.

**Figure 2:**
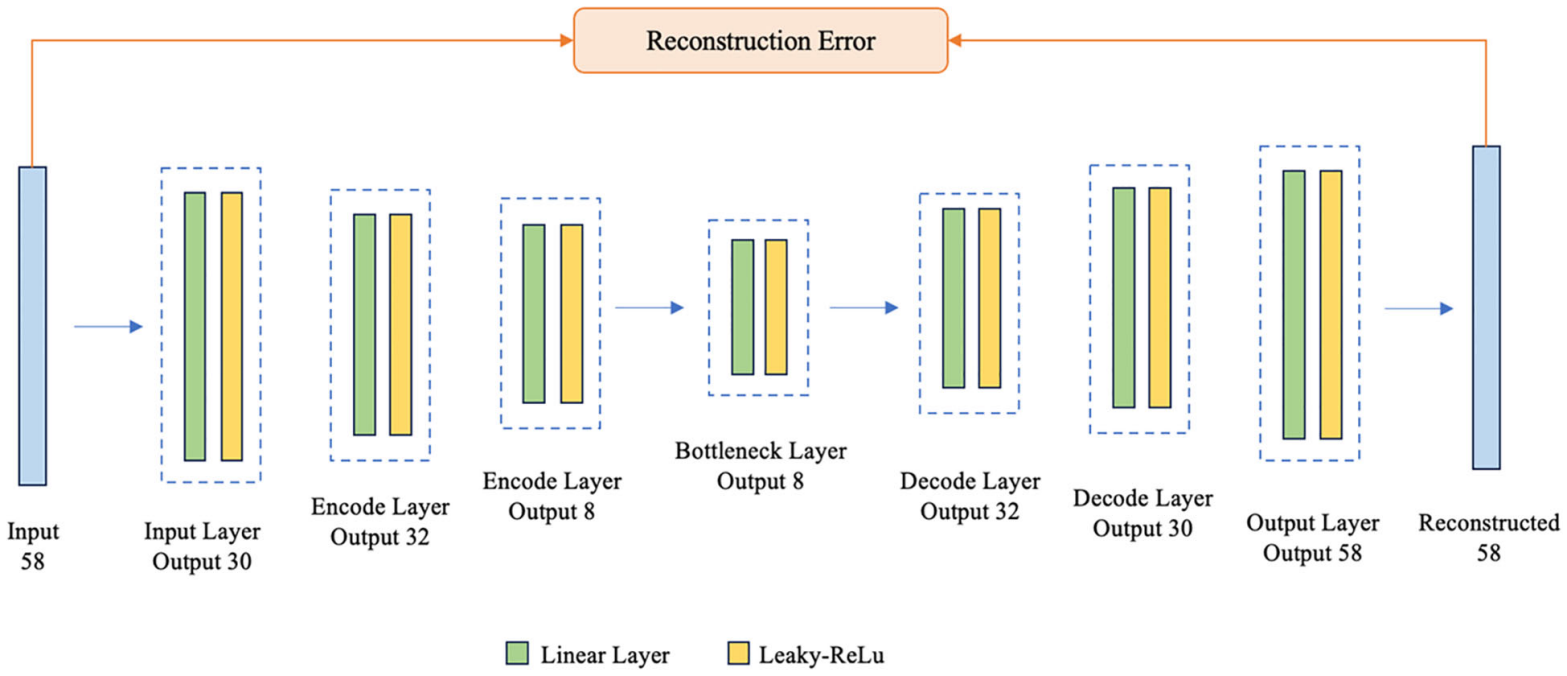
The autoencoder architecture.

In this study, the autoencoder (AE) is a deep-learning model for anomaly detection. It is an unsupervised learning model trained to minimize the reconstruction error between the input and its reconstructed counterpart. The AE architecture is illustrated in **Figure 2**. It is a feed-forward multilayer neural network with an encoder, a bottleneck, and a decoder. Both the encoder and the decoder contain multiple consecutive basic blocks that contain a pair of fully connected layer and activation function. The layer in the encoder gradually decreases in dimensionality and finally generates the layer with the lowest dimensionality, called latent code. The layer in the decoder gradually increases in dimensionality and recovers the latent code generated by the encoder to the same dimensionality as the network input layer.

To capture the reconstruction errors in enumerated and discrete variables after one-hot encoding, a binary cross-entropy (BCE) loss, *L_BCE_*, was used. In addition, to capture the reconstruction errors in continuous variables, the mean square error (MSE) loss, *L_MSE_*, was used. The objective of AE is the reconstruction loss, $L_{recon}=L_{BCE}+\lambda L_{MSE}$, i.e., the weighted sum of the two losses mentioned previously. Here, λ is the weight to balance the importance between *L_BCE_* and *L_MSE_* and then set to 0.99 in this study. This reconstruction error is the decision function of the detection model and also the anomaly score, i.e., *S(x)= L_recon_*.

A grid-search algorithm was employed to search for the best number of layers and neurons. As a result, the AE model has seven layers, including one input layer (30 neurons), one output layer (30 neurons), two encoding layers (32 and 8 neurons), two decoding layers (8 and 32 neurons), and one bottleneck layer (8 neurons). The Leaky ReLu activation function was used in the output layers. The optimization objective is the reconstruction error, i.e., *L_recon_*. The Adam optimizer was adopted with a learning rate 1e-3. The mini-batch wise Stochastic Gradient Descent (SGD) is used for training with a batch size of 128. The maximal number of training epochs is 3,000. Early stopping was used to find a sufficient number of training epochs and prevent overtraining the model. The network training was terminated if the reconstruction error on the validation data did not decrease for 20 consecutive epochs.

The distribution of reconstruction errors on the validation dataset was used to select the threshold. In this study, the threshold was chosen to ensure a 100% true positive rate (TPR or recall, i.e., judging a plan as an anomaly when it is actually anomalous) and as low as possible false positive rate (FPR, i.e., judging a plan as an anomaly when it is actually normal). Because radiation is harmful to patients, the plan review must ensure a high TPR, so that all anomalous plans are correctly detected, while maintaining a low FPR, so that as few normal plans as possible are incorrectly detected as anomalies. This means that the sensitivity of the detection model must be higher while the specificity of the detection model might be compromised. In a clinical setting, a 100% TPR may result in more wrongly detected anomalous plans and as such, increasing the workload for physicists. Since an anomalous plan may cause irreversible harm to a patient, the highest possible TPR is required.

### 2.4 Interpretation tool

**Figure 3:**
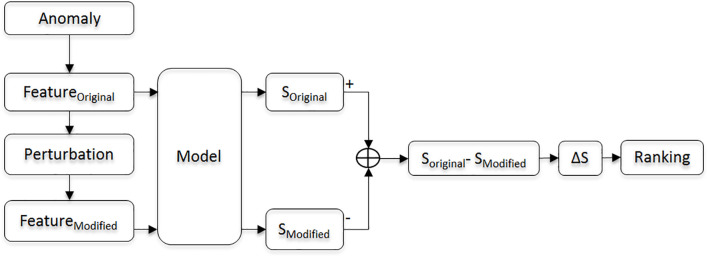
The workflow of the perturbation-based interpretation process.

The FPI method for the model interpretation process is illustrated in **Figure 3**. This involves modifying a single feature of an instance at a time and observing its effect on the result.

At first, the original value of the *i*-th feature of an instance is modified to the mean value *µ_i_*, which is calculated from normal plans in the training set, as given here:


$$X_{i}^{\prime }\left(j\right)=\{\begin{array}{ll} X_{i}\left(j\right),\; & \;if\;j\neq i\\ {µ}_{i},\; & if\;j=i \end{array}$$

Where *X_i_(j)* is the original instance and $X_{i\left(j\right)}^{\prime }$ is the modified instance when the values of the *i*-th feature is replaced by *µ_i_*. For the original and modified instances, *X* and *X*´, the anomaly scores $S_{i}^{o}=S\left(X\right)$ and $S_{i}^{m}=S\left(X^{\prime }\right)$ are calculated by the detection model for the *i*-th feature. The difference between $S_{i}^{o}$ and $S_{i}^{m}$ is then calculated as.


$$\Delta S_{i}=S_{i}^{o}-S_{i}^{m}.$$

A positive Δ*S_i_* signifies that the original feature value exacerbates the anomaly of an instance, while a negative Δ*S_i_* indicates that the original feature value contributes toward making the instance appear less abnormal (i.e., more normal). As Δ*S_i_* quantifies the effect of the i-th feature’s perturbation on the model’s output, it denotes the importance of the *i*-th feature on the given instance and the so-called importance score (IS). In the following, all features were sorted based on their Δ*S_i_* values. Furthermore, the Δ*S_i_* value across all anomalies were computed as the average feature importance score ( $\bar{\Delta S_{i}}$) and used to rank all features for a model.

### 2.5 Evaluation

The performance of the detection models was evaluated based on the receiver operating characteristic (ROC) curve and the area under the ROC curve (AUC). The ROC evaluates the ability of the model to distinguish between anomalous and normal plans when a preset threshold is changed. In addition, considering the highly unbalanced distribution of abnormal and normal classes in the dataset, the accuracy [(true positive + true negative)/(true positive + false positive+ true negative +false negative)], precision [true positive/(true positive + false positive)], and F1 score [2*precision*recall/(precision + recall), where recall = true positive/(true positive + false negative)] of the model were calculated to comprehensively evaluate performance.

In addition to the AE model, the five popular detection algorithms were tested, namely, local dispersion factor (LOF) (31), hierarchical density-based spatial clustering of applications with noise (HDBSCAN) (32), one-class support vector machine (OC-SVM) (33), principal component analysis (PCA) (34), and isolation forest (IF) (35). LOF is a density-based detection algorithm and HDBSCAN is a clustering-based algorithm. OC-SVM uses a single classification algorithm based on optimization algorithm. PCA calculates the reconstruction loss of linear mapping. IF operates through a recursive partitioning process, creating multiple decision trees that help identify anomalies.

## 3 Results

### 3.1 Detection models

**Figure 4:**
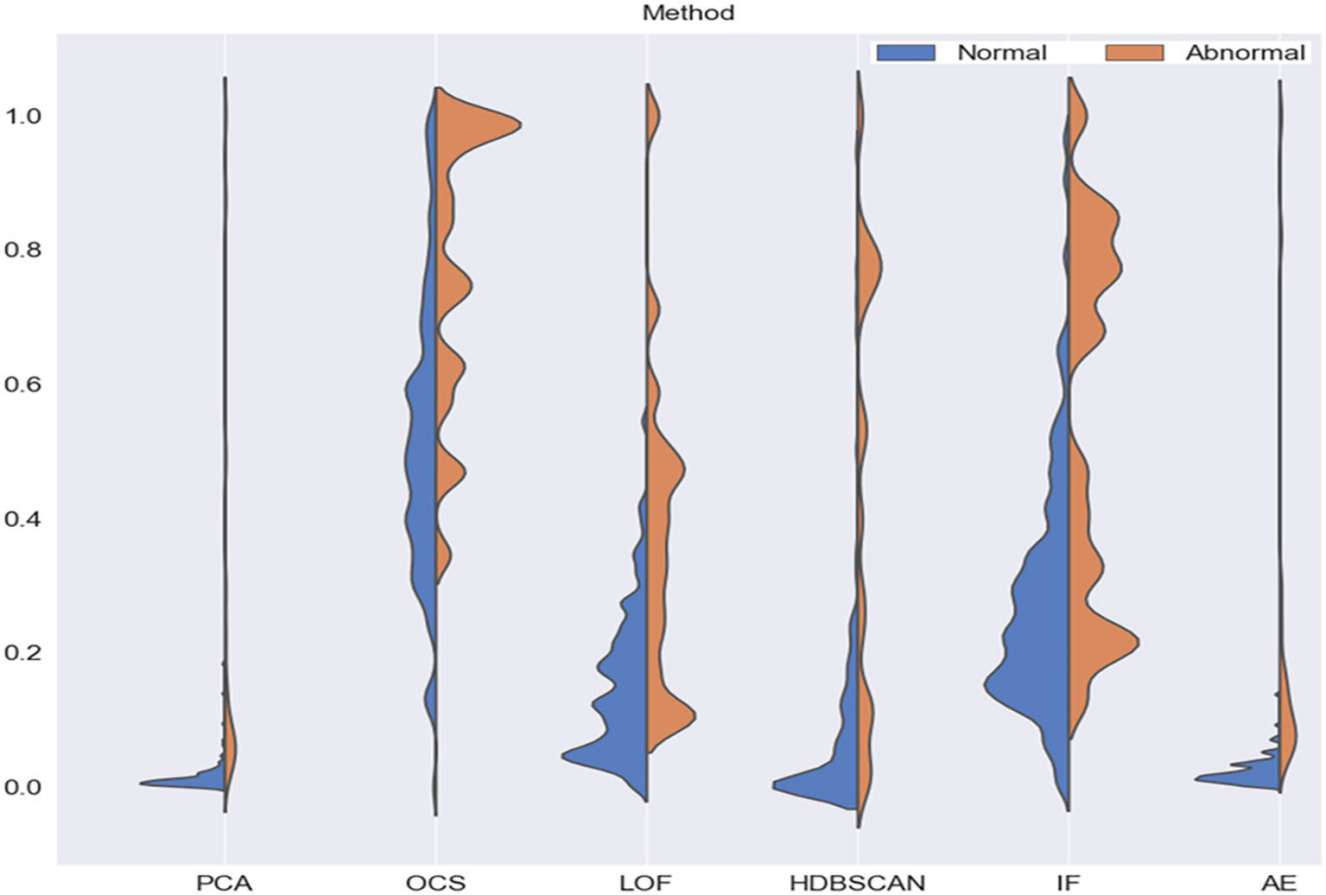
The reconstruction error distribution of all detection models.

To compare each detection model as fairly as possible, we used a grid-search algorithm to search for the parameters in each model that would achieve the best detection results. **Table 2** shows the anomaly detection performance of these detection models and their best hyper-parameters. AE achieved the best anomaly detection performance compared to the other selected classic methods. Based on the results of the five-fold cross-validation, the AUC, accuracy, precision, FPR, and F1 scores for the AE model were 0.98 ± 0.03, 0.91 ± 0.14, 0.61 ± 0.14, 0.10 ± 0.16, and 0.74 ± 0.13, respectively. The distributions of reconstruction errors for all detection models are shown in **Figure 4**. The x-axis shows the detection models and the y-axis shows the distributions of their normalized losses. The AE had the lowest overlap between the reconstruction error distributions of anomalous and normal plans.

**Table 2:** Comparison of the detection models.

Models	AUC	Accuracy	Precision	FPR	TPR	F1 score
AE (depth = 6)	0.98	0.91	0.61	0.10	1.00	0.74
LocalOutFactor (n_neighbor = 10)	0.81	0.46	0.19	0.61	1.00	0.32
HDBSCAN (min_cluster_size = 200)	0.84	0.46	0.20	0.60	1.00	0.33
OneClassSVM (nu = 0.001, gamma = 0.1)	0.80	0.30	0.16	0.79	1.00	0.27
PCA (n = 36)	0.96	0.81	0.42	0.20	1.00	0.58
IF	0.78	0.33	0.16	0.76	1.00	0.28

### 3.2 Interpretation methods

**Figure 5:**
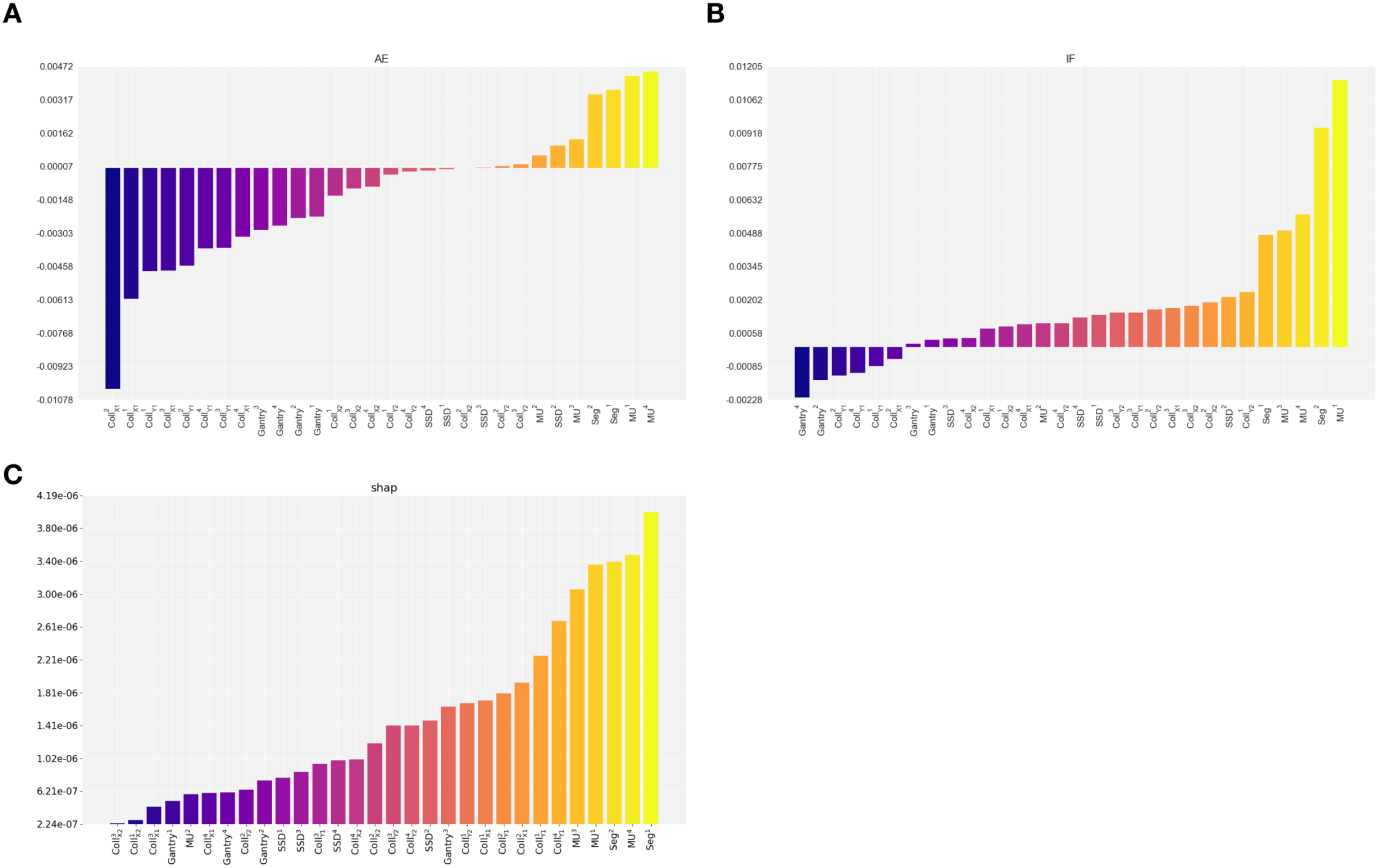
The average feature importance scores of all the features provided by three model interpretation methods: **(a)** Rank provided by FPI for AE. **(b)** Rank provided by local-DIFFI for IF. **(c)** Rank provided by SHAP for AE.

**Figure 6:**
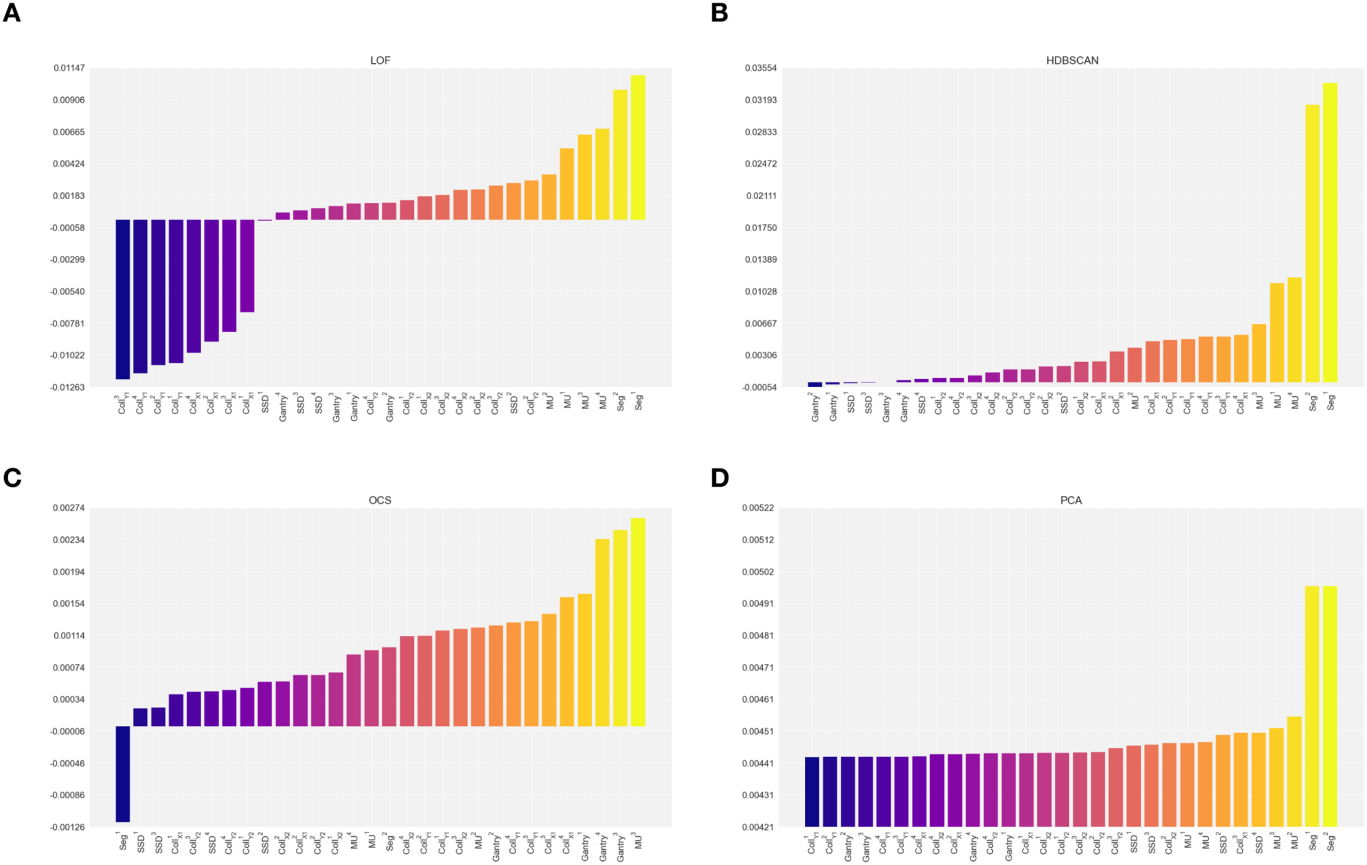
The average feature importance scores of all the features provided by FPI for the four classic detection models: **(a)** Rank provided by FPI for LOF. **(b)** Rank provided by FPI for HDBSCAN. **(c)** Rank provided by FPI for OC-SVM. **(d)** Rank provided by FPI for PCA.

The average feature importance scores, $\bar{\Delta S_{i}}$, provided by FPI for AE, local-DIFFI for IF, and SHAP for AE models are shown in **Figures 5a–c**, respectively. The features are displayed in ascending order (along the x-axis) according to their feature importance scores (shown in the y-axis). The cold color (blue) and warm color (yellow) indicate the small and large values of the importance scores, respectively. The figure shows that the top five features of both models are the same.

( $\bar{\Delta S_{i}}$) provided by FPI for the four classic detection models are also shown in **Figure 6**.

**Figure 7:**
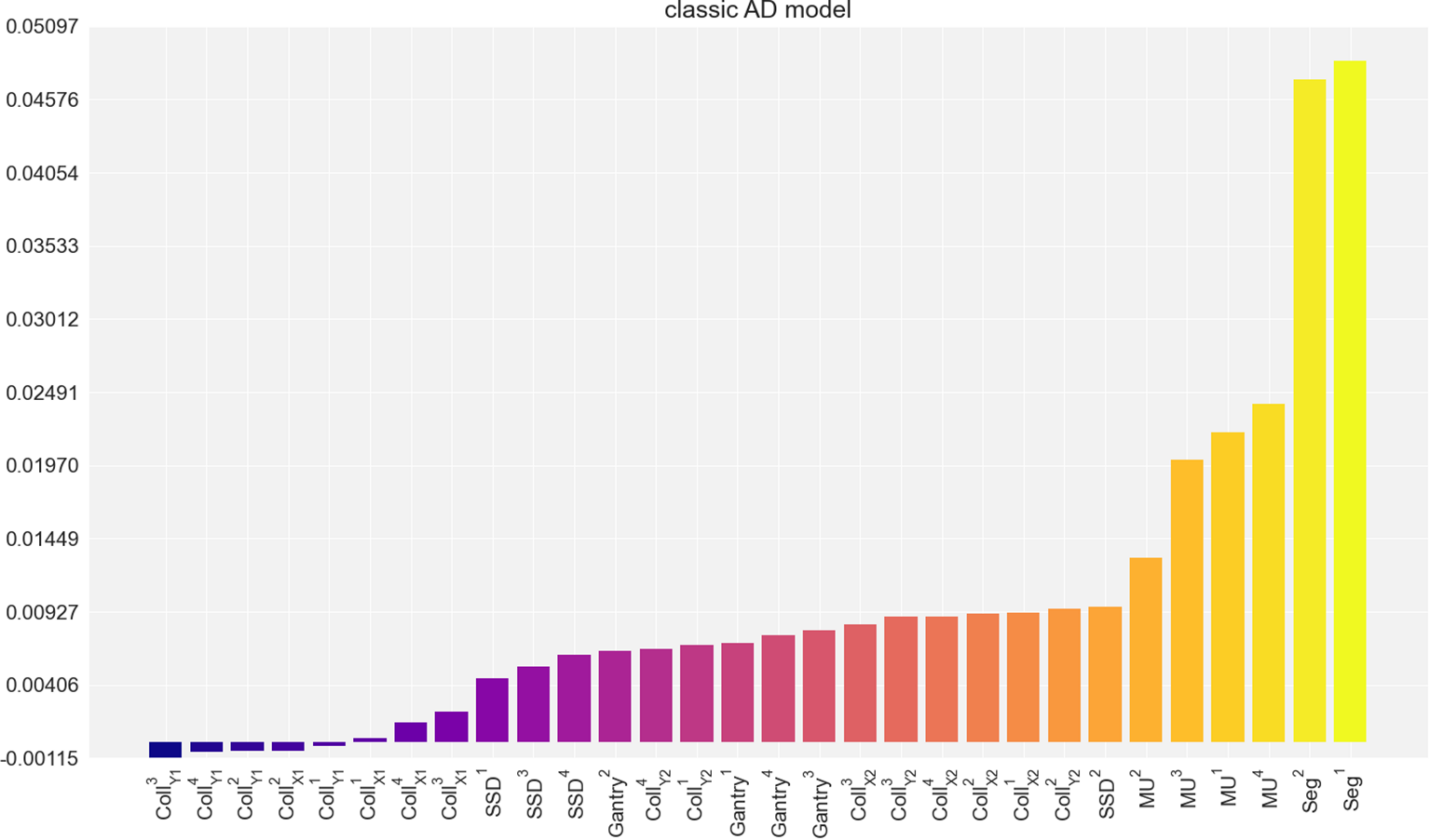
The mean rank of the feature importance scores provided by FPI for the four classic detection models.

The values of ( $\bar{\Delta S_{i}}$) across ranks, provided by FPI for the four classic models in **Figure 6**, are also averaged and shown in **Figure 7**. This mean rank represents the overall feature importance for the four classic detection models.

The top five important features provided by FPI for AE, local-DIFFI for IF, SHAP for AE, and FPI for the four classic models are summarized in **Table 3**. *Seg*^1^, *Seg*^2^, *MU*^1^, *MU*^3^, and *MU*^4^ are commonly shared by the results provided by FPI, local-DIFFI, and SHAP methods. Although the locations of the five features are different in ranks as shown in **Table 3**, all these features with a larger impact on the model output are selected by the interpretation methods.

**Table 3:** The top five features selected by the interpretation tool.

Rank	FIP for the AE model		Local-DIFFI for the IF model		SHAP for the AE model		FIP for the classic models	
	Feature	IS	Feature	IS	Feature	IS	Feature	IS
1	*MU* ^4^	0.00449	*MU* ^1^	0.0114	*Seg* ^1^	3.99e-06	*Seg* ^1^	0.0485
2	*MU* ^1^	0.00427	*Seg* ^2^	0.00942	*MU* ^4^	3.47e-06	*Seg* ^2^	0.0471
3	*Seg* ^1^	0.00362	*MU* ^4^	0.00568	*Seg* ^2^	3.39e-06	*MU* ^4^	0.0240
4	*Seg* ^2^	0.00343	*MU* ^3^	0.00499	*MU* ^1^	3.35e-06	*MU* ^1^	0.0220
5	*MU* ^3^	0.00133	*Seg* ^1^	0.00480	*MU* ^3^	3.06e-06	*MU* ^3^	0.0201

IS, importance score.

## 4 Discussion

This study evaluated the performance of the AE model and interpretation tool in an automated anomaly detection of a physics plan review. For comparison, various classic anomaly detection models were tested. The results show that AE is superior to the classic detection model in several aspects. The AE method employs a nonlinear activation function in the encoder/decoder, allowing the neural network to arbitrarily approximate any nonlinear function. This allows the network to learn more complex mapping relationships between high-dimensional space and low-dimensional space, to better fit the distribution of normal data, and thus, find abnormal data with a very small percentage through the network. AE is a relatively simple deep-learning network applied in the field of physics plan review and the more sophisticated networks could be introduced to further improve the current performance of anomaly detection.

The effectiveness of the FPI method was validated on local-DIIFI and SHAP methods. As the reference method, local-DIFFI was more acceptable and understandable from a clinical perspective. The difference it presented when compared to the FPI method was not significant. As shown in **Table 3**, the top five features were the same for the three methods, which indicated the effectiveness and reliability of the FPI method. Additionally, the FPI method can be applied to most detection models while local-DIFFI was only specified for the IF model. The FPI method provides valuable insights into machine-learning models but further evaluation is needed to ascertain definitive advantages in clinical applications.

Anomalous plans detected by the plan review process are rare because most acceptable for clinical treatment and differ only slightly from normal plans. The imbalanced distribution of the normal and anomalous plans could result in poor predictive performance of the detection models, specifically for those supervised learning-based models. However, our detection model was based on unsupervised learning and trained on all normal plans. The effect of imbalanced data on the proposed model would be minor. Including external datasets from the other institutes could improve robustness of the results and validate the generalizability of the proposed model. This will be our next step. The challenge is that the scanning and treatment planning procedures and protocols in multiple institutes could vary considerably. The consistency of multi-institutional datasets was not warranted. In our institute, an automatic program was used to perform the initial checking task. This program followed the checklist provided by AAPM TG275 and was customized to our clinical workflow. Most of the plan parameters (simulation image, mechanical setting, dosimetrical output, etc.) can be automatically inspected by this program. This program can find errors by matching parameters with the preset thresholds but can hardly detect outliers. For these outliers, the advanced deep-learning model should be employed. For demonstration purposes, only a few core parameters and a simple treatment modality were chosen. Incorporating advanced plan features and treatment modalities, such as dose distributions, multileaf collimator parameters, and plan complexity indices could improve the model’s performance but would require greater effort in data pre-processing, model configuration, and training. Future work should explore the use of more advanced deep-learning networks and detection strategies in a real clinical setting.

The current application only focuses on IMRT plans and could be extended to other complicated treatment modalities, such as volumetric modulated arc therapy (VMAT), which is popular in current radiotherapy. VMAT consists of hundreds of radiation fields with uniform intensities and delivered with gantry rotation. During VMAT delivery, the motions of gantry and multi-leaf collimator are synchronized to ensure the higher quality of dose conformity. The checklist not only includes static parameters such as monitor units (MUs) and apertures, but also motion factors such as gantry and leaf motions. As a solution, it would be feasible to extract the aperture maps of all control points in a VMAT and export them to a learning model for anomaly detection. This will help to identify apertures at certain control points that have uncommon shapes and positions. The aperture map can also be used jointly with other plan features, such as dose map, to build a more comprehensive anomaly detection model for plan review in VMAT.

The application of AE and a perturbation-based interpretation tool in the field of physics plan review of radiotherapy is promising. However, it can be improved in several aspects. First, the detection of anomalous plans is difficult and may happen once in several hundreds of plans. This could result in a highly imbalanced dataset and cause potential over-fitting of the learning model. Synthesizing anomalous data using a generative deep-learning model to compensate the minority class could be the solution and will be investigated in our future research. Second, AE is a commonly used deep-learning model that is simple but less specific. Incorporating new learning modules, such as attention-based transformers, may further improve the current AE models. This will extend our work to a higher performance level. Third, although the method provides interpretable outputs, transforming these outputs into clinical practice is difficult. Training tools and public datasets may be necessary to allow clinicians to validate the model’s logic and build trust in the technology.

## 5 Conclusion

An approach for anomaly detection and model interpretation in physics plan review was evaluated in this study. The FPI method improved interpretability by providing clinical users with important information on the risk factors in a radiotherapy plan. The proposed approach demonstrated effectiveness and adaptability comparable to those of established techniques. While the proposed approach reveals promising results, future work should validate its applicability across diverse treatment sites and radiotherapy modalities.

## Figures and Tables

**Figure 8:**
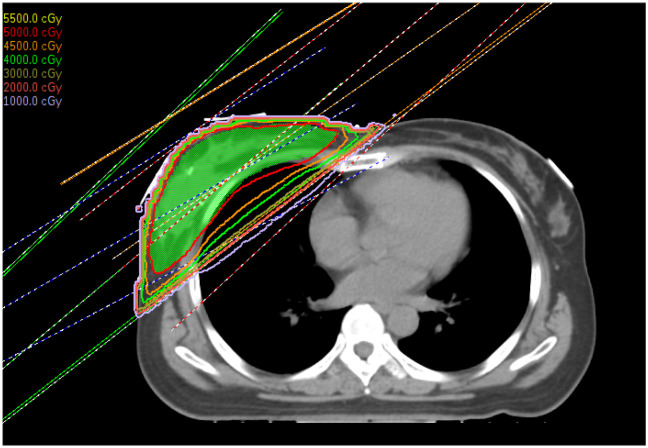
The illustration of a typical beam setting in a hybrid plan.

## Data Availability

The original contributions presented in the study are included in the article/supplementary material. Further inquiries can be directed to the corresponding authors.

## APPENDIX 1

The description of features.

All treatment plans in this study are hybrid plans that consist of two tangent (conformal) fields and two modulated fields. For the two modulated fields, the jaw positions were fixed for all segments. Therefore, there was one constant jaw position for all segments in the modulated field. The tangent fields and modulated fields in a plan were given 80% and 20% prescription doses, respectively. The prescription dose was 50 Gy (200 cGy×25 fractions). The typical beam setting for a hybrid plan is shown in **Figure 8**. The green and organ lines represent the two tangent fields, while the blue and red lines represent the two modulated fields. The 30 features extracted from each hybrid plan are listed in **Table 4**.

**Table 4:** The detailed descriptions of the features of a hybrid plan.

Features	Categories	Description	Type	Unit
Seg^1^	Segment	The number of segments of modulated field 1	integer	number
Seg^2^	Segment	The number of segments of modulated field 2	integer	number
SSD^1^	SSD	Source-to-skin distance of tangent field 1	float	Cm
SSD^2^	SSD	Source-to-skin distance of tangent field 2	float	Cm
SSD^3^	SSD	Source-to-skin distance of modulated field 1	float	Cm
SSD^4^	SSD	Source-to-skin distance of modulated field 2	float	Cm
Coll^1^_x1_	Coll_x1_	Collimator position in the x1 direction of tangent field 1	float	Cm
Coll^2^_x1_	Coll_x1_	Collimator position in the x1 direction of tangent field 2	float	cm
Coll^3^_x1_	Coll_x1_	Collimator position in the x1 direction of modulated field 1	float	cm
Coll^4^_x1_	Coll_x1_	Collimator position in the x1 direction of modulated field 1	float	cm
Coll^1^_x2_	Coll_x2_	Collimator position in the x2 direction of tangent field 1	float	cm
Coll^2^_x2_	Coll_x2_	Collimator position in the x2 direction of tangent field 2	float	cm
Coll^3^_x2_	Coll_x2_	Collimator position in the x2 direction of modulated field 1	float	cm
Coll^4^_x2_	Coll_x2_	Collimator position in the x2 direction of modulated field 2	float	cm
Coll^1^_y1_	Coll_y1_	Collimator position in the y1 direction of tangent field 1	float	cm
Coll^2^_y1_	Coll_y1_	Collimator position in the y1 direction of tangent field 2	float	cm
Coll^3^_y1_	Coll_y1_	Collimator position in the y1 direction of modulated field 1	float	cm
Coll^4^_y1_	Coll_y1_	Collimator position in the y1 direction of modulated field 2	float	cm
Coll^1^_y2_	Coll_y2_	Collimator position in the y2 direction of tangent field 1	float	cm
Coll^2^_y2_	Coll_y2_	Collimator position in the y2 direction of tangent field 2	float	cm
Coll^3^_y2_	Coll_y2_	Collimator position in the y2 direction of modulated field 1	float	cm
Coll^4^_y2_	Coll_y2_	Collimator position in the y2 direction of modulated field 2	float	cm
Gantry^1^	Gantry	Gantry angle of tangent field 1	integer	degree
Gantry^2^	Gantry	Gantry angle of tangent field 2	integer	degree
Gantry^3^	Gantry	Gantry angle of modulated field 1	integer	degree
Gantry^4^	Gantry	Gantry angle of modulated field 2	integer	degree
MU^1^	MU	Monitor unit of tangent field 1	float	MU
MU^2^	MU	Monitor unit of tangent field 2	float	MU
MU^3^	MU	Monitor unit of modulated field 1	float	MU
MU^4^	MU	Monitor unit of modulated field 2	float	MU
